# Evaluation of BioFire® FilmArray® Pneumonia Panel in Bronchoalveolar Lavage Samples From Immunocompromised Patients With Suspected Pneumonia

**DOI:** 10.7759/cureus.38024

**Published:** 2023-04-23

**Authors:** Tony Li-Geng, Fainareti N Zervou, Maria Aguero-Rosenfeld, Ioannis M Zacharioudakis

**Affiliations:** 1 Department of Medicine, New York University (NYU) Grossman School of Medicine, New York City, USA; 2 Department of Medicine, Division of Infectious Diseases and Immunology, New York University (NYU) Grossman School of Medicine, New York City, USA; 3 Department of Pathology, New York University (NYU) Grossman School of Medicine, New York City, USA

**Keywords:** antimicrobial stewardship, microbiology, community aquired pneumonia, transplant infections, immunocompromised hosts, bronchoalveolar lavage (bal), filmarray pneumonia panel, multiplex pcr

## Abstract

Objectives

Immunocompromised patients, specifically those with solid organ transplants or cancer on chemotherapy, are at particularly high risk of severe pneumonia and opportunistic infections. In select patients, bronchoalveolar lavage (BAL) is performed to provide high-quality samples for analysis. We compare BioFire® FilmArray® Pneumonia Panel (BioFire Diagnostics, Salt Lake City, Utah, United States), a multiplex polymerase chain reaction (PCR) assay, with standard of care diagnostics in BAL samples from immunocompromised patients to identify opportunities for this test to affect clinical decision making.

Methods

Patients hospitalized with pneumonia based on clinical and radiographic findings who underwent evaluation with bronchoscopy between May 2019 to January 2020 were reviewed. Among those patients undergoing bronchoscopy, those who were immunocompromised were selected for inclusion in the study. BAL specimens submitted to the microbiology laboratory were chosen based on as part of the internal validation of the panel in comparison with sputum culture at our hospitals. We compared the outcomes of the multiplex PCR assay with traditional culture methods and evaluated the role of PCR assay in de-escalating antimicrobial therapy.

Results

Twenty-four patients were identified for testing with the multiplex PCR assay. Of the 24 patients, 16 were immunocompromised, all with solid or hematological malignancy or a history of organ transplant. Seventeen individual BAL samples from the 16 patients were reviewed. BAL culture results and the multiplex PCR assay were in agreement in 13 samples (76.5%). In four cases, the multiplex PCR assay identified a possible causative pathogen not detected by standard workup. The median time to de-escalation of antimicrobials was three days (interquartile range (IQR) 2-4) from the day of collection of the BAL samples.

Conclusions

Studies have established the additive role of multiplex PCR testing in addition to traditional diagnostic tools like sputum culture in diagnosing the etiology of pneumonia. Limited data exist specifically looking at immunocompromised patients, in whom a timely and accurate diagnosis is particularly important. There is a potential benefit for performing multiplex PCR assays as an additive diagnostic tool in BAL samples for these patients.

## Introduction

Pneumonia has been the most common cause of hospitalization due to infectious disease in the United States and the second most common cause of hospitalization overall [1-2]. Although the 2019 Infectious Diseases Society of America (IDSA)/American Thoracic Society (ATS) guidelines do not recommend routine microbiologic workup in patients hospitalized with community-acquired pneumonia [3], they do recommend a tiered approach to diagnostic evaluation with non-invasive and invasive microbiologic workup in immunocompromised hosts, such as bone marrow and solid organ transplant recipients [4]. In the context of the coronavirus disease 2019 (COVID-19) pandemic, the need for rapid and sensitive diagnostics to evaluate for bacterial superinfection is even more crucial, especially in mechanically ventilated patients, both to guide pathogen-directed therapy and to also avoid over-utilization of antibiotics [5]. In this study, we compare the results of BioFire® FilmArray® Pneumonia Panel (BioFire Diagnostics, Salt Lake City, Utah, United States), a multiplex polymerase chain reaction (PCR) assay with those of the standard microbiologic workup on bronchoalveolar lavage (BAL) specimens collected from immunocompromised patients with evidence of pneumonia. We identify opportunities for this test to affect clinical decision-making in this high-risk patient population.

## Materials and methods

Study setting and population

The study was conducted at New York University Langone Health (NYULH), an academic medical center composed of three hospitals in the greater New York metropolitan area, United States. From May 2019 to January 2020, BAL specimens submitted to the microbiology laboratory for bacterial cultures from patients with clinical and radiographic signs of pneumonia were selected after chart review by an infectious disease physician. This was done as a part of a quality improvement project for the internal validation of the pneumonia multiplex PCR panel at our hospitals. Included samples then had multiplex PCR performed for comparison with standard cultures. Clinical criteria included documented fever > 101^o^ Fahrenheit, new/worsening productive cough or increase in respiratory secretions in intubated patients, pleuritic chest pain, or new/worsening dyspnea or hypoxia, defined as documented blood oxygen saturation <92%. Radiographic criteria included findings on chest imaging of airspace opacity, lobar consolidation, or interstitial opacities, new or worsening compared to the available baseline.

During the study period, 24 patients with signs of pneumonia received bronchoscopy and BAL. Each BAL sample was tested with both traditional culture methods as well as multiplex PCR, although the clinical team was provided only with results from standard microbiologic testing and not results from the multiplex PCR. In further reviewing the patients, 16 were noted to be immunocompromised and due to institutional interest in the diagnostic workup in this population, these patients were selected to be included in assessing the role of multiplex PCR in immunocompromised patients. Patients with solid or hematologic malignancy receiving chemotherapy, with bone marrow or solid organ transplant on immunosuppression, receiving chronic immune-suppressing medications, with human immunodeficiency virus, CD4 count <200 cells/mm^3^, or inherited immunodeficiency were included. Because the purpose of this study was for quality improvement, institutional review board approval was not required for this project.

Biofire® FilmArray® Pneumonia Panel

The BioFire® FilmArray® Pneumonia Panel is an FDA-cleared multiplex PCR assay for use on sputum and BAL samples with a turn-around time of about one hour [6]. It is a combined real-time PCR with a lab-in-a-pouch system that automatically performs all steps of the assay from nucleic acid extraction to nested multiplex PCR and data analysis [7]. It semi-quantitatively detects 15 bacterial agents (*Acinetobacter calcoaceticus-baumannii* complex, *Enterobacter cloacae* complex, *Escherichia coli*, *Haemophilus influenzae*, *Klebsiella aerogenes*, *Klebsiella oxytoca*, *Klebsiella pneumoniae* group, *Moraxella catarrhalis*, *Proteus spp*., *Pseudomonas aeruginosa*, *Serratia marcescens*, *Staphylococcus aureus*, *Streptococcus agalactiae, Streptococcus pneumoniae, and Streptococcus pyogenes*) to assist with differentiation between colonization and true infection [8,9] and qualitatively detects three atypical bacterial (*Chlamydia pneumoniae, Legionella pneumophila, and Mycoplasma pneumoniae*) and eight viral pathogens (adenovirus, coronavirus, human metapneumovirus, human rhinovirus/enterovirus, influenza A virus, influenza B virus, parainfluenza virus, and respiratory syncytial virus). Antimicrobial resistance genes including the methicillin resistance genes mecA/C and MREJ, the carbapenemases: *Klebsiella pneumoniae* carbapenemase (KPC), New-Delhi metallo-beta-lactamase (NDM), Oxa-48-like, Verona integron‒encoded metallo-beta-lactamase (VIM), active-on-imipenem (IMP), and the extended-spectrum beta-lactamase CTX-M are also detected.

Outcomes of interest

The primary outcome of interest was the percentage of BAL samples for which a microbiologic diagnosis was obtained using the multiplex PCR assay compared with the results of standard of care microbiologic workup.

A secondary outcome of interest was to investigate the potential of the multiplex PCR assay to lead to a change of antimicrobial therapy by detecting pathogens that were not covered by empiric antimicrobials, by detecting pathogens that allowed for de-escalation of therapy, or by providing reasons to consider expansion of the diagnostic workup.

Data extraction and analysis

We reviewed electronic medical records and extracted the demographic characteristics and comorbidities of the patients included in our analysis. We reviewed all the results of the diagnostic workup done as per routine standard of care, including BAL bacterial cultures, *L. pneumophila* Serogroup I and *S. pneumoniae* urinary antigens, *M. pneumoniae* serologies, multiplex PCR panel, and viral cultures. Data regarding time to culture result, time to any change of antimicrobial therapy from date of bronchoscopy, change of immunosuppression, and final microbiologic diagnosis were also collected. Estimates are presented as median with interquartile range (IQR).

## Results

A total of 24 patients were selected for testing with the multiplex PCR assay based on the criteria described in the Methods. On retrospective review, 17 samples were from 16 patients with solid or hematological malignancy or a history of solid organ transplant (SOT). One patient had two independent presentations that required assessment with BAL and, therefore, had two independent evaluations with multiplex PCR. The median age of patients was 71 years (IQR 59.5-73.5) and 10 were male (62.5%). Ten patients were SOT recipients, including five with heart transplants, three with lung transplants, one with a kidney transplant, and one with a combined kidney-liver transplant. Five SOT patients received their transplant during the same admission when the BAL sample was collected. The average time from transplant to BAL collection was five months. All SOT patients were on an immunosuppressive regimen, the most common of which included mycophenolate mofetil and tacrolimus (eight patients, 80%), as well as prednisone (six patients, 60%). Patients with malignancy carried a range of diagnoses, including non-small cell lung cancer, melanoma, Hodgkin’s lymphoma, and acute myeloid leukemia. Two chemotherapy regimens included biologics; pembrolizumab for non-small cell lung cancer and ipilimumab and nivolumab for melanoma. 

Twelve patients (66.7%) were diagnosed with pneumonia on presentation to the emergency room, while four were diagnosed with pneumonia >72 hours after admission. The most common lung imaging findings were multifocal opacities, ground-glass appearance, and focal consolidation for five, five, and four patients respectively. The median length of stay was 22 days (IQR 6.5-48) with nine patients requiring intubation and five patients requiring extracorporeal membrane oxygenation. Two patients died during their hospitalization.

**Figure 1:**
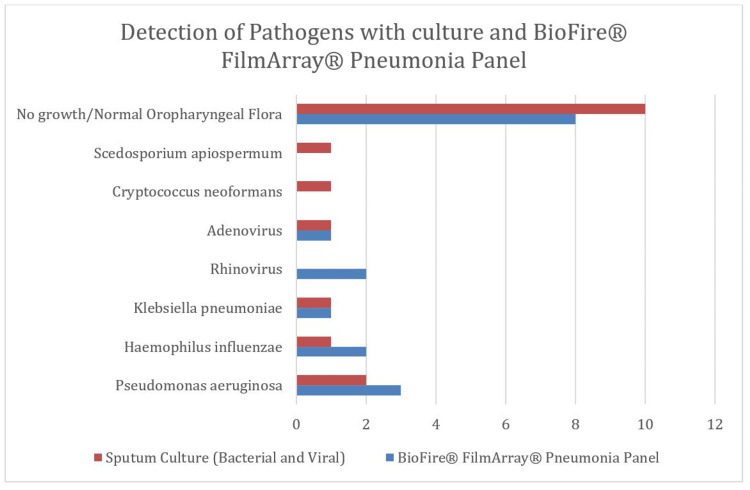
Summary of Performance of the BioFire® FilmArray® Pneumonia Panel* in Comparison to Sputum Cultures *BioFire Diagnostics, Salt Lake City, Utah, United States

Based on traditional culture methods, seven BAL samples (41.2%) identified an infectious etiology of pneumonia. These included two *P. aeruginosa* (11.9%), one *K. pneumoniae* (5.9%), one*H. influenzae* (5.9%), one *C. neoformans* (5.9%), one *S. apiospermum* (5.9%), and one adenovirus (6.3%). Seven BAL samples (41.2%) had no growth in the culture and three BAL samples (17.6%) grew normal oropharyngeal flora (NOF), which was deemed to not be clinically significant. *L. pneumophila* Serogroup I and *S. pneumoniae* urinary antigens and *M. pneumoniae* serologies were performed on 15 patients and all were negative. Using the multiplex PCR assay, nine BAL samples identified an infectious etiology of pneumonia. These included three *P. aeruginosa* (17.6%), two *H. influenzae* (11.9%), one *K. pneumoniae* (5.9%), two rhinovirus (11.9%), and one adenovirus (5.9%). Traditional culture results and multiplex PCR assay were in agreement in 13 samples (76.5%). In four cases, the multiplex PCR assay identified a possible causative pathogen not detected by standard workup: two cases of rhinovirus, one of *P. aeruginosa*, and one of *H. influenza*. Two patients had culture growth of organisms that are not tested in the multiplex PCR assay: *C. neoformans* and *S. apiospermum*. In agreement with the culture results, no resistance genes were detected. The results and performance of traditional culture methods in comparison to multiplex PCR assay are described in Table 1 and Figure 1.

**Table 1:** Results of BAL Culture and BioFire® FilmArray® Pneumonia Panel* M: male; F: female; K: 1000; NOF: normal oropharyngeal flora; BAL: bronchoalveolar lavage *BioFire Diagnostics, Salt Lake City, Utah, United States

Age	Sex	Transplant or Malignancy Type	BAL Bacterial Culture (Colony Forming Units)	BAL Viral Culture	BAL BioFire® FilmArray® Pneumonia Panel	Chest Imaging Findings	Antimicrobials at time of Pneumonia Diagnosis	Days to De-escalation from Day of BAL Culture Result	Diagnosis
70	M	Deceased Donor Kidney	Cryptococcus neoformans	No growth	None	Patchy nodular infiltrate with consolidation and air bronchograms, small ground-glass opacity	Pip-tazo, levofloxacin	1	*Cryptococcus *pneumonia
56	M	Combined Liver Kidney	70K *Klebsiella pneumoniae*	No growth	10^7^ *Klebsiella pneumoniae*	Bilateral interstitial opacities	Vancomycin, pip-tazo, azithromycin, micafungin	2	*Klebsiella pneumoniae *pneumonia
71	M	Bilateral Lung	10K *Scedosporium apiospermum*	No growth	Rhinovirus	Extensive multifocal consolidation, ground-glass opacities, tree-in-bud nodularity	Vancomycin, cefepime, azithromycin	8	*Scedosporium apiospermum* pneumonia
19	F	Bilateral Lung	>10K *Pseudomonas aeruginosa*	No growth	10^7 ^*Pseudomonas aeruginosa*	Extensive bilateral bronchiectasis with slight interval increase in consolidation	Pip-tazo, tobramycin nebulizer	1	*Pseudomonas aeruginosa* pneumonia
19	F	Bilateral Lung	No growth	No growth	10^5^*Pseudomonas aeruginosa*	Bilateral airspace opacities	Vancomycin, pip-tazo, tobramycin nebulizer	1	*Pseudomonas aeruginosa *pneumonia
57	F	Orthotopic Heart	>100K *Pseumonas aeruginosa*	No growth	10^7 ^*Pseudomonas aeruginosa*	New mucoid impaction with complete right lower lobe consolidation	Vancomycin, rifabutin, ethambutol, doxycycline	1	*Pseudomonas aeruginosa *pneumonia
72	F	Orthotopic Heart	No growth	No growth	None	Extensive mixed interstitial and airspace process	Vancomycin, cefepime	4	Diffuse Alveolar Hemorrhage
74	M	Orthotopic Heart	100K *Haemophilus influenzae*	No growth	10^7^*Haemophilus influenzae*	Opacification of left hemithorax	Vancomycin, levofloxacin, meropenem	2	^29^ *Haemophilus influenzae* pneumonia
36	M	Orthotopic Heart	Few NOF	No growth	None	Worsening bilateral lung opacities, severe diffuse alveolar damage	Vancomycin, meropenem, micafungin	14	Pulmonary edema
65	M	Orthotopic Heart	3K NOF	No growth	None	Cavitary lesion of right lower lobe	Vancomycin, pip-tazo, isavuconazole	4	Aspiration pneumonia
73	M	Orthotopic Heart	No growth	No growth	None	Multifocal nodular and confluent consolidations, bilateral pleural effusions	Pip-tazo, azithromycin	1	Sirolimus toxicity
79	F	Non-small Cell Lung Cancer	500 NOF	No growth	None	Diffuse groundglass opacity	Ceftriaxone, azithromycin	3	*Pneumocystis* pneumonia
62	M	Hodgkin's Lymphoma	No growth	Adenovirus	Adenovirus	Large consolidation, tree-in-bud nodes, and opacities	Vancomycin, pip-tazo, doxycycline	2	Adenovirus pneumonia
71	F	Ductal Carcinoma in Situ	No growth	No growth	None	Diffuse hazy groundglass opacity	Ceftriaxone, azithromycin	3	*Pneumocystis* pneumonia pulmonary embolism
71	M	Multiple Myeloma	No growth	No growth	None	Multifocal patchy consolidation	Vancomycin, pip-tazo, azithromycin	6	Without diagnosis
74	M	Acute Myeloid Leukemia	No growth	Not performed	Rhinovirus	Multifocal groundglass opacities	Vancomycin, pip-tazo	7	Rhinovirus pneumonia
86	F	Melanoma	No growth	No growth	10^6^ Haemophilus Influenzae	Moderate multifocal patchy, reticulonodular, and groundglass opacities	Ceftriaxone, azithromycin	3	Bacterial pneumonia

The median time to the final result of sputum culture with speciation and sensitivities was three days (IQR 1-5). Because viral culture was a send-out test, it consistently took 14 days to provide results. Among samples with positive results and agreement between multiplex PCR assay and routine culture, the mean difference in time to positive result was 2.2 days. The initial antimicrobial regimen at time of diagnosis of pneumonia was highly varied, as several patients had alternative sources of infection or extensive workups prior to bronchoscopy. Empiric antibiotic regimens most frequently included vancomycin in 11 cases (64.7%), piperacillin-tazobactam in nine cases (52.9%), and azithromycin in seven cases (41.2%). Antifungals, including micafungin and isavuconazole, were started empirically in three cases (17.6%). Five (29.4%) cases had more targeted antibiotic therapies selected within 24 hours of initial BAL culture results being reported. Eight (72.7%) SOT patients had at least one component of their immunosuppression regimen held due to severe pneumonia. In seven cases, mycophenolate mofetil was held and in seven cases, prednisone was held. In one case, sirolimus was held due to sirolimus-related toxicity. Twelve (70.6%) hospitalizations carried the diagnosis of culture-proven infectious pneumonia. Non-infectious causes included diffuse alveolar hemorrhage, pulmonary edema, and sirolimus toxicity.

## Discussion

We examined the correlation between the results of traditional culture and BioFire® FilmArray® Pneumonia Panel and the potential additive yield of the multiplex PCR assay in reaching a microbiologic diagnosis in immunocompromised patients undergoing BAL with clinical and radiographic findings of pneumonia. We found that when comparing traditional bacterial and viral culture methods with multiplex PCR assay, there was 76.5% agreement. In the four cases with incongruent results between traditional culture methods and the multiplex PCR assay, the latter identified two samples with bacteria and two with rhinovirus. The two patients with bacterial pneumonia had *H. influenzae* and *P. aeruginosa* detected on multiplex PCR assay and both saw clinical response to empiric antibiotics, with de-escalation of antibiotics after one to three days. Meanwhile, the two patients with rhinovirus detected received an empiric course of seven to eight days of broad-spectrum antibiotics before de-escalation. In these two situations, the results of the multiplex PCR assay could have supported earlier antibiotic de-escalation by the clinician. There were no cases of bacteria or virus detected by standard of care diagnostics that were not detected by the multiplex PCR assay. One case of *C. neoformans* and one case of *S. apiospermum* were detected and clinically significant but not detected by multiplex PCR assay, as it is not designed to detect fungal pathogens. 

Data on BAL yield in immunocompromised patients is limited. One assessment in heart transplant patients with suspected lower respiratory tract infection found that BAL findings established specific diagnoses in only 41% of cases [10]. In studies comparing multiplex PCR assay of BAL fluid with traditional culture, it has been suggested that multiplex PCR like LightCycler SeptiFast® (Roche Diagnostics, Mannheim, Germany) can provide higher identification rates of pathogens [11-12]. BioFire® FilmArray® Pneumonia Panel has added benefit in its ability to identify viruses and resistance genes as well as to provide semi-quantitative results [13]. Quantitative results are potentially additive by assessing the concentration of bacteria present, thereby theoretically differentiating colonization from true infection. Meanwhile, data on virus culture compared to multiple PCR in BAL samples has demonstrated modern techniques are more sensitive than traditional culture methods in the identification of respiratory viruses [14]. However, more studies are needed to evaluate the direct impact of the BioFire® FilmArray® Pneumonia Panel on utilization and cost-effectiveness.

Finalized traditional culture methods on average require 42 hours longer than multiplex PCR assays to result [15], suggesting that anti-microbial de-escalation could be further expedited with multiplex PCR assays, depending on laboratory workflow. Among our samples with positive traditional culture results, the mean difference in time to positive results between the two methods was 2.2 days. Additionally, five (29.4%) of cases had more targeted antibiotic therapies selected within 24 hours of results of BAL traditional culture results. This suggests that even in immunocompromised patients, clinicians respond to culture data. Considering the multiplex PCR assay had 100% sensitivity in our cohort in comparison to traditional cultures, clinicians may feel more confident to target therapies earlier with the additional data. Due to the design of this study as a quality improvement project, further prospective data with larger sample sizes would be needed to demonstrate external validity.

Antimicrobial stewardship is important in immunocompromised patients who are likely to be empirically started on broad-spectrum antimicrobials and therefore at risk for infections due to multidrug-resistant organisms and *Clostridioides difficile* infection [16-17]. Additionally, antimicrobial stewardship and data-driven treatment changes are particularly useful in immunocompromised patients who are at risk for less common etiologies of pneumonia like *C. neoformans* pneumonia, *Pneumocystis* pneumonia (PCP), *Aspergillus* pneumonia, and cytomegalovirus (CMV) pneumonia, as these patients are also at risk of non-infectious causes like pneumonitis, diffuse alveolar hemorrhage, and pulmonary edema [18-20]. In this cohort, three patients were ultimately diagnosed with non-infectious causes. Sirolimus toxicity was diagnosed based on findings on bronchoscopy and despite collecting BAL cultures, clinicians did not await culture results to discontinue antimicrobials due. Diffuse alveolar hemorrhage was diagnosed with direct visualization during bronchoscopy and antimicrobials were discontinued shortly after. The patient diagnosed with pulmonary edema was continued on broad-spectrum antimicrobial coverage, including antifungal, for 14 days. It is difficult to comment on whether negative multiplex PCR assay results would have affected the decision to continue antimicrobials in this patient with a new heart transplant, but negative results may have led to stronger consideration of pulmonary edema as the primary diagnosis. Although the patient with non-small cell lung cancer on pembrolizumab was diagnosed with bacterial pneumonia, the possibility of pembrolizumab-associated pneumonitis was strongly considered in the differential. Early microbiological data for these patients can mean an earlier decision to continue antimicrobials or to initiate steroids for drug-associated pneumonitis. In immunocompromised patients, the high rate of microbiological diagnosis by multiplex PCR assay can help clinicians to be more confident to target antibiotic therapy or seek an alternative diagnosis [21].

Clinicians should keep in mind that opportunistic pathogens causing pneumonia in immunocompromised hosts such as *Pneumocystis jiroveci*, *C. neoformans*, CMV, and *Toxoplasma gondii *are not included in the panel. This is particularly relevant in immunocompromised patients. In our cohort, one patient was diagnosed with *C. neoformans* pneumonia using traditional culture methods; however, the multiplex PCR assay is not designed to detect this pathogen. Despite two patients being diagnosed with PCP, neither had routine culture results, including fungal culture, to confirm the diagnosis. They were empirically treated for PCP as they had elevated lactate dehydrogenase (LDH), positive 1,3-beta-d-glucan, and bilateral groundglass opacities on imaging. There may be additive benefit to PCR testing for *P. jiroveci *in BAL samples [22-23], but the BioFire® FilmArray® Pneumonia Panel is unable to detect this pathogen, along with other opportunistic pathogens that can be seen in immunocompromised patients.

This study is limited by the small sample size of immunocompromised patients with BAL samples from a single center and its design as a quality improvement project. While designed with inclusion criteria, the study served as an internal validation project and allowed for a large range of patient illness severity and clinical courses. Some patients without severe pneumonia were only hospitalized for several days while other patients received solid organ transplantation during the same admission and had month-long hospitalizations. Decisions to de-escalate antimicrobials are affected by clinical severity, especially when testing is unrevealing. This study was performed to validate the use of pneumonia panels and results did not directly guide clinical practice. These clinical decisions have been further complicated by the emergence of severe acute respiratory syndrome coronavirus 2 (SARS-CoV-2) as a novel infectious etiology of pneumonia. Validation of data from this study was completed before the first case of COVID-19 was detected in March 2020 in New York City.

The data regarding diagnostic testing in immunocompromised patients is not robust, but the American Society of Transplantation does strongly recommend nucleic acid testing methods on BAL fluid when obtained [4]. Multiplex PCR assays provide rapid and accurate diagnostic data [24]. Previous work has suggested multiplex PCR assays substantially increase the rate of microbiologic diagnosis and provide opportunities for antimicrobial optimization in patients with pneumonia [21]. However, limited literature is available on their utility on BAL fluid samples or in immunosuppressed patients. Available literature notes that molecular diagnostic testing shortens time to diagnosis but cannot replace standard of care culture methods in lung transplant patients undergoing bronchoscopy [25].

## Conclusions

Our quality improvement project suggests that there is a potential for BioFire® FilmArray® Pneumonia Panel could be an important diagnostic tool in BAL samples from immunosuppressed patients with suspected pneumonia. The small number of specimens and the lack of randomization of patients selected requires further studies to determine the additive benefit of the BioFire® FilmArray® Pneumonia Panel. Nevertheless, the data was sufficient for our institution to adapt this assay for clinical use for immunocompromised patients. Further studies should evaluate clinical outcome data and cost-effectiveness for the incorporation of the routine use of multiplex PCR assays in immunocompromised patients.
